# P50 Five-year review of epidemiology of invasive group A streptococcal infections with bacteraemia in a tertiary care centre in the UK

**DOI:** 10.1093/jacamr/dlaf118.057

**Published:** 2025-07-14

**Authors:** Adam Pattinson, Chinagozi Edwin, Karthika Priya Subbaraj, G I D Dushyanthie, A D Athukorala

**Affiliations:** Microbiology Department, University Hospital Coventry and Warwickshire (UHCW), UK; Microbiology Department, University Hospital Coventry and Warwickshire (UHCW), UK; Microbiology Department, University Hospital Coventry and Warwickshire (UHCW), UK; Microbiology Department, University Hospital Coventry and Warwickshire (UHCW), UK; Microbiology Department, University Hospital Coventry and Warwickshire (UHCW), UK

## Abstract

**Background:**

The national rate of Invasive Group A Streptococcal (iGAS) infection reporting remains stable, however, West Midlands—the region we serve—is highlighted as one of the areas with highest notification rates in the UK.^1^ Despite a considerable number of iGAS infections that our team is involved in managing annually, we have not reviewed the demographics, spectrum, severity, outcome or the antibiotic sensitivity data for iGAS infections at our centre. This study was carried out to assess details with a view to better understand iGAS infections within our patient population in order to improve care.

**Methods:**

Blood culture specimens positive for *Streptococcus pyogenes* (GAS) at our centre from 1 January 2019 to 31 December 2023 and the respective clinical records for patients were retrospectively reviewed.

**Results:**

One hundred and eighty-six cases of GAS bacteraemias were identified and were equally distributed among male (49.5%) and female gender (50.5%). The age distribution analysis indicated that the highest rate of GAS bacteraemia occurred in the age range of 61–90 years followed by 31–50 years and 1–10 years, respectively. The lowest rate occurred in the <1 year age range followed by 11–30 years age range. The commonest focus of infection was skin and soft tissue (56.45%), followed by respiratory tract (18.8%). No focus of infection was identified in 11.8% cases. Thirty-nine distinct *emm* types were identified among study isolates and *emm* 1.0 was the most prevalent (28.5%), followed by *emm* 89.0 (6.98%) and *emm* 12.0 (6.45%). Notably, *emm* 1.0 was the most common strain among patients who passed away by day 30 (50% of fatal cases). Disc diffusion susceptibility testing was performed for amoxicillin, erythromycin, clindamycin, doxycycline, linezolid, penicillin, rifampicin, teicoplanin and vancomycin in accordance with the EUCAST guidance published each year. Susceptibility analysis of all isolates revealed universal susceptibility to penicillin, vancomycin, teicoplanin and linezolid. Resistance to clindamycin which is used as an adjunct therapy for anti-toxin effect in management of iGAS infections was very low at 2.15%. The rates of resistance to doxycycline and erythromycin were 11.83% and 8.6% respectively. Thirty day mortality was approximately 18%, rising to 21% at 90 days, with no sex-based difference (male 53% versus female 47%). However, extremes of age was a significant risk factor for 30 day mortality: infants (<1 year) and the elderly (>90 years) had the highest mortality rates at 50% and 40%, respectively. Notably, 50% of GAS isolates associated with 30 day mortality were *emm* type 1.0.

**Conclusions:**

Age was most strongly associated with mortality. Antibiotic resistance rates were low and was not a determinant of mortality. *emm* 1 was increasingly represented in patients that passed away compared to those that survived. Ongoing work is reviewing the relationship between *emm* type and source of infection, and prescribing practices at our tertiary care centre.

## References

[1] Group A Streptococcal Infections: First Update on Seasonal Activity in England, 2024 to 2025. https://www.gov.uk/government/publications/group-a-streptococcal-infections-seasonal-activity-in-england-2024-to-2025/group-a-streptococcal-infections-first-update-on-seasonal-activity-in-england-2024to-2025

